# EGFRvIII expression triggers a metabolic dependency and therapeutic vulnerability sensitive to autophagy inhibition

**DOI:** 10.1080/15548627.2017.1409926

**Published:** 2018-01-29

**Authors:** Barry Jutten, Tom G. Keulers, Hanneke J. M. Peeters, Marco B. E. Schaaf, Kim G. M. Savelkouls, Inge Compter, Ruud Clarijs, Olaf E. M. G. Schijns, Linda Ackermans, Onno P. M. Teernstra, Marijke I. Zonneveld, Resi M. E. Colaris, Ludwig Dubois, Marc A. Vooijs, Johan Bussink, Julio Sotelo, Jan Theys, Guido Lammering, Kasper M. A. Rouschop

**Affiliations:** a Department of Radiotherapy, GROW – School for Oncology and Developmental Biology, Maastricht University Medical Centre+, Maastricht, The Netherlands; b Department of Clincial Pathology, Zuyderland MC, Sittard-Geleen, The Netherlands; c Department of Neurosurgery, Maastricht University Medical Centre; d Department of Radiation Oncology, Radboud University Medical Center, Nijmegen, The Netherlands; e Neuroimmunology and Neuro-Oncology Unit, National Institute of Neurology and Neurosurgery, Mexico City, Mexico; f Heinrich- Heine University Duesseldorf, Germany; g Department of Radiation Oncology (MAASTRO Clinic), GROW School for Oncology and Developmental Biology, Maastricht University Medical Centre+, The Netherlands

**Keywords:** Autophagy, chloroquine, EGFR, EGFRvIII, glioblastoma, hypoxia, metabolic stress radiotherapy, starvation

## Abstract

Expression of EGFRvIII is frequently observed in glioblastoma and is associated with increased cellular proliferation, enhanced tolerance to metabolic stresses, accelerated tumor growth, therapy resistance and poor prognosis. We observed that expression of EGFRvIII elevates the activation of macroautophagy/autophagy during starvation and hypoxia and explored the underlying mechanism and consequence. Autophagy was inhibited (genetically or pharmacologically) and its consequence for tolerance to metabolic stress and its therapeutic potential in (EGFRvIII^+^) glioblastoma was assessed in cellular systems, (patient derived) tumor xenopgrafts and glioblastoma patients. Autophagy inhibition abrogated the enhanced proliferation and survival advantage of EGFRvIII^+^ cells during stress conditions, decreased tumor hypoxia and delayed tumor growth in EGFRvIII^+^ tumors. These effects can be attributed to the supporting role of autophagy in meeting the high metabolic demand of EGFRvIII^+^ cells. As hypoxic tumor cells greatly contribute to therapy resistance, autophagy inhibition revokes the radioresistant phenotype of EGFRvIII^+^ tumors in (patient derived) xenograft tumors. In line with these findings, retrospective analysis of glioblastoma patients indicated that chloroquine treatment improves survival of all glioblastoma patients, but patients with EGFRvIII^+^ glioblastoma benefited most. Our findings disclose the unique autophagy dependency of EGFRvIII^+^ glioblastoma as a therapeutic opportunity. Chloroquine treatment may therefore be considered as an additional treatment strategy for glioblastoma patients and can reverse the worse prognosis of patients with EGFRvIII^+^ glioblastoma.

## Abbreviations

ATG7: autophagy-related 7
CQ: chloroquine
EGFP: enhanced green fluorescent protein
EGFR: epidermal growth factor receptor
EGFRvIII: epidermal growth factor receptor variant 3
EGFRvIII^+^: EGFRvIII positive
EGFRvIII^−^: EGFRvIII negative
GBM: glioblastoma multiforme/glioblastoma
MAP1LC3B/LC3B: microtubule-associated protein 1 light chain 3 beta
PDX: patient derived xenograft
RRAS/RAS type GTPase family: related RAS viral (r-ras) oncogene
shRNA: short hairpin ribonucleic acid
ULK1: unc-51 like kinase 1

## Introduction

EGFR (epidermal growth factor receptor) is a transmembrane glycoprotein and one of 4 members of the ERBB family of tyrosine kinase receptors. Binding of one of its natural ligands results in homo- or heterodimerization of the receptor, autophosphorylation and subsequent activation of signal transduction pathways. These pathways are involved in regulating cellular proliferation, differentiation, and survival.^1^^,^^2^ Although present in normal cells, EGFR is overexpressed in a variety of tumors and has been associated with poor prognosis and decreased survival.^3^ EGFR activation also plays a role in resistance to chemotherapy and radiation treatment in tumor cells.^4^^,^^5^

In cancer, EGFR is often mutated, leading to enhanced or sustained receptor signaling. One of the most common variants of EGFR is an exon 2–7 deletion mutant, EGFRvIII (also termed ΔEGFR or de2-7EGFR).^6–9^ This deletion results in a truncated receptor that lacks 267 amino acids in the extracellular binding domain. This deletion leads to important functional changes; the receptor is unable to bind its ligands, but becomes constitutively active, resulting in uncontrolled pro-oncogenic effects and thereby promotes neoplastic transformation and tumorigenicity.^10–13^ EGFRvIII expression is observed in several malignancies, including glioblastoma (GBM), non-small lung cell carcinoma, breast cancer, prostate cancer and head and neck cancer^6^^,^^8^^,^^9^ and is associated with poor prognosis.^14^ In GBM patients with amplification of the *EGFR* gene, the overall prevalence of EGFRvIII is 50–60%^15^ and has been reported to contribute to tumor stem cell maintenance.^16–18^ Expression of EGFRvIII greatly enhances GBM tumorigenicity in vivo^19^^,^^20^ and stimulates cell invasion in vitro and in vivo.^21^^,^^22^

The tumor microenvironment is characterized by extreme heterogeneities in nutrient supply and oxygenation that arise primarily due to a poorly developed and/or functioning vascular network. Gradients of oxygenation exist around individual perfused vessels and range from normal values (∼5% O_2_) near the vessel wall to anoxia in peri-necrotic regions. Transient changes in blood flow also lead to strong temporal changes in oxygenation within specific tumor regions.^23^ The percentage of viable hypoxic tumor cells within individual tumors with otherwise similar clinical features varies tremendously among patients^24^ and is clinically important because high levels of tumor hypoxia correlate with poor prognosis and a more aggressive phenotype.^24-26^ The source of the variability in hypoxia among different tumors is likely due to the acquisition of changes that drive increased hypoxia tolerance.^27^ Strikingly, EGFRvIII expression in vivo is rapidly lost when tumor cells are cultured in vitro under nutrient-rich conditions.^28^ This observation suggests that in the tumor microenvironment, which is characterized by heterogeneities in nutrient supply and oxygenation, EGFRvIII-expressing tumor cells have a survival advantage, a finding supported by pre-clinical evidence.^12^^,^^13^

To survive starvation and hypoxia, cells respond by upregulation of autophagy (Greek for ‘self-eating’).^29^^,^^30^ A number of recent studies have demonstrated that human tumor cell lines induce autophagy when exposed in vitro to hypoxia and/or metabolic stress,^31–33^ which mediates both selective and bulk degradation of proteins, cytoplasmic content, and organelles, and enables a cell to recycle constituents and provide itself with the necessary nutrients to maintain energy levels, protein synthesis and essential metabolic processes.^34^

Based on our observations that EGFRvIII expression results in increased autophagy activation in metabolically challenged cells, we hypothesized that the increased survival and growth of EGFRvIII-expressing cells and tumors during stressful conditions is supported by autophagy. Targeting autophagy could therefore be a potential tool to lower tolerance to metabolic stresses and increase cell killing of EGFRvIII-expressing GBM cells.

## Results

### EGFRvIII-expressing cells are highly dependent on autophagy during starvation

**Figure 1.**
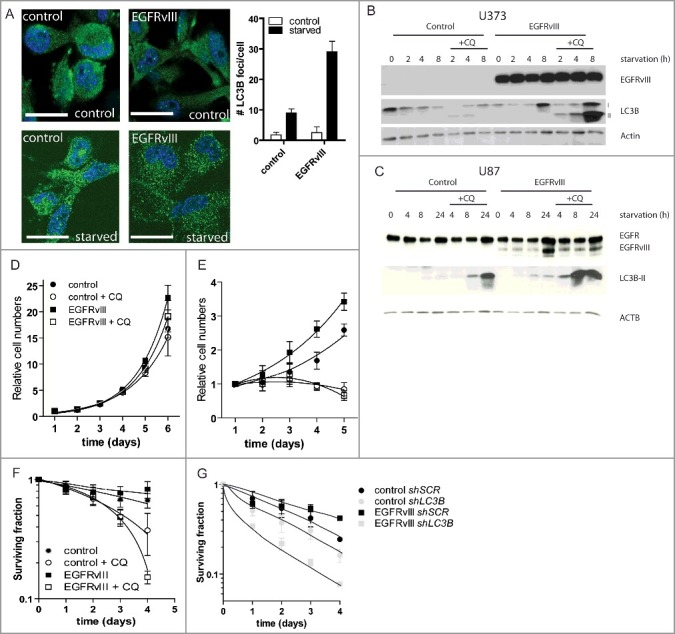
EGFRvIII-expressing cells display increased autophagy activation and dependence during serum-starved conditions. (**A**) EGFRvIII-expressing cells display more and larger autophagosomes during starvation as assessed by immunofluorescence (nuclei [DAPI] in blue, LC3B in green). Scale bars: 10 μm. Immunoblot analysis of control and EGFRvIII-expressing cells reveal elevated autophagic flux during serum-starved conditions in EGFRvIII-expressing U373 (**B**) and U87-MG (**C**) cells. Proliferation curves under normal (**D**) and serum-starved (**E**) conditions show that CQ (5 µg/ml) does not influence growth under normal conditions, but can abrogate the growth advantage of EGFRvIII-expressing cells during starvation. n = 3, mean ± SEM. Clonogenic survival assessment after serum starvation with or without addition of CQ (**F**) or expression of a shRNA targeting *LC3B* (**G**) indicate that the elevated survival advantage of EGFRvIII-expressing cells is autophagy dependent. n = 3, mean ± SEM. SCR, scrambled control.

EGFR is often overexpressed or mutated in diverse cancer types and especially in glioblastoma. One of the most commonly found mutations in EGFR is the constitutive active deletion variant EGFRvIII, which contributes to increased intrinsic radioresistance but also increases tolerance to microenvironmental factors that contribute to therapy resistance (e.g., hypoxia).^24–26^^,^^35^^,^^36^ The tumor microenvironment is characterized by the presence of regions that are low in oxygen (hypoxia) and nutrient supply. Previously we and others have shown that cells deprived of nutrients or oxygen rapidly activate autophagy.^31–33^^,^^37^ Considering the high proliferation rate and nutritional demand of EGFRvIII-expressing cells,^38^ we explored autophagic activity in EGFRvIII-expressing cells. Immunohistochemical analysis during serum-starved conditions revealed more and larger autophagosomes in the EGFRvIII-expressing cells compared to the control cells (Fig. 1A), suggesting changes in autophagic activity.

The gold standard in autophagy activity determination, is the measurement of autophagic flux. In short, during autophagy LC3B is partially degraded. Blocking lysosomal function prevents this degradation and the accumulation of this protein during these conditions reflects autophagic activity (flux). To achieve lysosomal inhibition, cells were exposed to nutrient deprivation in the absence and presence of the lysosomotropic drug, chloroquine (CQ, 5 µg/ml). A faster and more pronounced induction of autophagy, as indicated by the accumulation of LC3B-II, was observed in the EGFRvIII-expressing U373 (Fig. 1B) and U87 MG cells (Fig. 1C). Exposure to afatinib, an irreversible tyrosine kinase inhibitor, decreases autophagic flux in EGFRvIII-expressing cells in a dose-dependent manner, indicating that the increased autophagic flux in EGFRvIII-expressing cells during starvation was EGFRvIII-activity dependent (Fig. S1A).

To determine if the higher autophagic flux contributed to growth advantages of EGFRvIII-expressing cells, cellular proliferation during normal and starvation conditions was determined. CQ addition had no significant effect on proliferation of either control or EGFRvIII-expressing cells during normal culture conditions (Fig. 1D), indicating that the used concentration was nontoxic at this dose. During starvation, EGFRvIII-expressing cells proliferated faster compared to control cells (Fig. 1E, p < 0.05). This growth advantage was abrogated by the addition of CQ to the culture medium. To assess whether autophagy inhibition alters survival of EGFRvIII-expressing cells, clonogenic survival after serum deprivation was determined. Serum deprivation did not significantly change clonogenic survival of control or EGFRvIII-expressing cells (Fig. 1F). However, CQ addition resulted in increased cell death of EGFRvIII-expressing cells (p < 0.05 Control +CQ versus EGFRvIII + CQ at t = 4). Although CQ has been widely used as an inhibitor of autophagy, its effects are not solely restricted to the inhibition of autophagy. To confirm if the observed effects were dependent on autophagy, we engineered cells expressing a doxycycline-inducible shRNA directed against *LC3B*. In agreement with an autophagy dependence, targeting *LC3B* sensitized cells to serum deprivation. In line with our pharmacological data, this effect was most pronounced in EGFRvIII-expressing cells (Fig. 1G, p < 0.05).

Taken together, these data indicate that EGFRvIII-expressing cells activate autophagy to a higher extent and are highly dependent on autophagy for survival in response to serum deprivation.

## EGFRvIII-expressing cells depend more on autophagy during hypoxia

**Figure 2.**
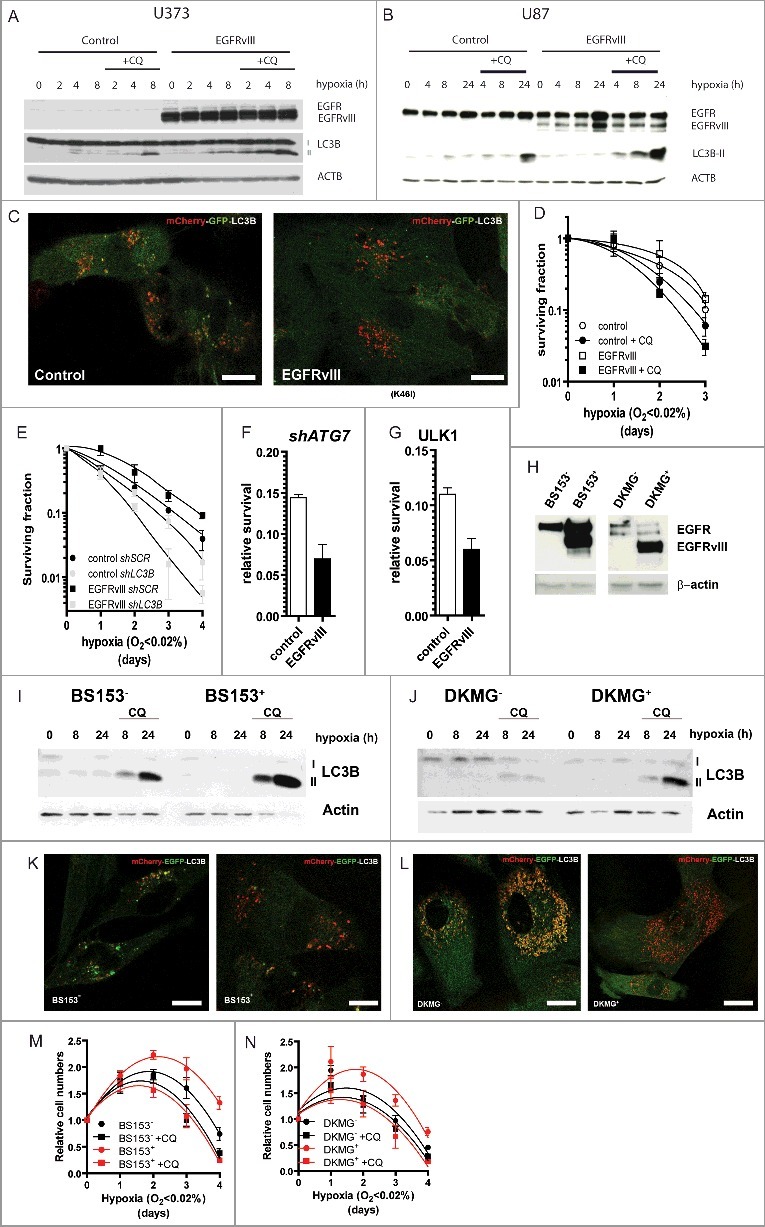
Inhibition of the elevated autophagic response in EGFRvIII-expressing cells abrogates their survival advantage during hypoxia. Immunoblot analysis indicates elevated autophagy activation in EGFRvIII-expressing U373 (A) and U87 (B) cells during hypoxia. (C) Tandem GFP-mCherry-LC3B expression indicates elevated autophagic flux in EGFRvIII^+^ cells during hypoxia (24 h O_2_<0.02%). Scale bars: 2 μm. Autophagy inhibition either through CQ addition (5 µg/ml) (D) or shRNA expression directed against *LC3B* (E) abrogates the survival advantage of EGFRvIII-expressing cells as assessed by clonogenic survival assay. n = 3, mean ± SEM (F) Targeting *ATG7* through expression of a shRNA (G) or ULK1 through expression of dominant negative ULK1^K46I^ results in loss of survival advantage of EGFRvIII^+^ cells as determined by clonogenic survival assay after 48 h hypoxia (O_2_<0.02%). n = 3, mean ± SEM (H) EGFR and ERGFRvIII expression of BS153 and DKMG cell populations. Autophagic flux determination in (I) BS153 and (J) DKMG cells during hypoxia. Tandem GFP-mCherry-LC3B expression indicates elevated autophagic flux in EGFRvIII^+^ (K) BS153 and (L) DKMG cells during hypoxia (24 h O_2_<0.02%). Scale bars: 2 μm. Autophagy inhibition through CQ addition (5 µg/ml) abrogates the survival advantage of EGFRvIII^+^ (M) BS153 and (N) DKMG cells as assessed by crystal violet staining after hypoxia exposure. n = 3, mean ± SEM.

To assess if the enhanced autophagic response was restricted to serum deprivation or also applies to other types of metabolic stress present within the tumor microenvironment, we assessed the autophagic response and dependency on autophagy during hypoxic conditions. Similar to serum deprivation, EGFRvIII^+^ cells displayed an enhanced autophagic flux compared to the control cells as indicated by faster LC3B-II accumulation in the presence of CQ (5 µg/ml) in U373 (Fig. 2A) and U87 MG cells (Fig. 2B). This was further confirmed using tandem mCherry-EGFP-LC3B expression in U373 cells. Although no clear differences could be observed during normal culture conditions (not shown), after 16 h hypoxia exposure, EGFRvIII^+^ cells displayed mostly red foci whereas EGFRvIII^−^ cells also displayed green/yellow foci (Fig. 2C). These data indicate faster processing of autophagosomes in EGFRvIII^+^ cells and support the flux analysis results seen by immunoblot.

To assess the dependency on autophagy for the survival of EGFRvIII-expressing cells, clonogenic survival after hypoxia exposure was determined in the presence of CQ (Fig. 2D) or after expressing a short hairpin that targets *LC3B* (Fig. 2E), or *ATG7* (Fig. 2F) or after overexpression of dominant negative ULK1^K46I^ (Fig. 2G). As reported previously,^12^ EGFRvIII-expressing cells displayed a survival advantage in comparison to control cells, yet EGFRvIII-expressing cells were sensitized to hypoxia (O_2_ < 0.02%) more than control cells in the presence of CQ or after targeting autophagy genetically (Fig. 2D and E, both p < 0.05 at t = 3 and/or −4; Fig. 2F and G, p < 0.05).

In addition to analysis of cells that ectopically express EGFRvIII, we explored the autophagy dependence of glioblastoma cells that endogenously express EGFRvIII. EGFRvIII-enriched populations were obtained after cell sorting.^39^ This way BS153 and DKMG isogenic cell populations have been obtained that lack EGFRvIII expression or are highly enriched for EGFRvIII expression (Fig. 2H). Also endogenous EGFRvIII^+^ cells displayed enhanced autophagic flux during hypoxia in BS153 (Fig. 2I) and DKMG (Fig. 2J) isogenic cell lines. This was further confirmed using mCherry-EGFP-LC3B expression in these cells. EGFRvIII^+^ cells displayed mostly mCherry-positive foci whereas EGFRvIII^−^ also displayed yellow/green foci, suggesting a more rapid turnover of autophagosomes in EGFRvIII^+^ BS153 (Fig. 2K) and DKMG (Fig. 2L) cells. Due to their migratory character both BS153 and DKMG cells failed to form colonies. Therefore, the sensitivity of these cells to CQ (5 µg/ml) addition during hypoxia was assessed by chrystal violet staining after exposure to hypoxia. During ambient air incubation, this concentration of CQ did not influence cell proliferation (Fig. S1B and C). As observed in ectopically expressing EGFRvIII^+^ cells, endogenously expressing EGFRvIII^+^ cells displayed a survival advantage during hypoxia in BS153 (Fig. 2M) and DKMG (Fig. 2N) cells. This survival advantage was fully abrogated in the presence of CQ. These data indicate that cells are highly dependent on autophagy for survival during hypoxia after ectopic, but also endogenous, expression of EGFRvIII.

### EGFRvIII-expressing xenografts depend on autophagy for accelerated regrowth after therapy

**Figure 3.**
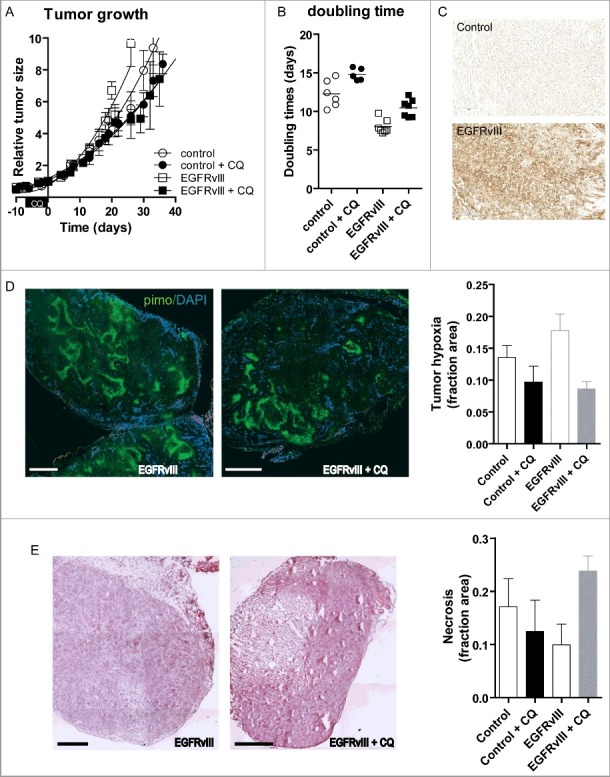
CQ treatment reduces tumor growth and tumor hypoxia and increases tumor necrosis in EGFRvIII-expressing tumors. (A) Growth curves of control U373 xenografts (open circles, n = 6) or treated from d −7 to 0 with CQ (60 mg/kg) (filled circles, n = 5) and EGFRvIII-expressing U373 xenografts (open squares, n = 7) treated with CQ (filled squares, n = 7). (B) Doubling times of the corresponding tumors. (C) Immunohistochemical staining for EGFRvIII on control (upper panel) and EGFRvIII-expressing xenografts (lower panel). Scale bars: 2 mm. (D) (Left panel) Micrographs of pimonidazole-DAPI staining prior to and after 7 d CQ treatment on EGFRvIII-expressing tumors. (Right panel) Quantification of the tumor hypoxic fraction (control n = 6, control + CQ n = 6, EGFRvIII n = 5, EGFRvIII + CQ n = 5). (E) (Left panel) Micrographs of hematoxylin and eosin staining prior to and after 7 d CQ treatment on EGFRvIII-expressing tumors. (Right panel) Quantification of tumor necrosis (control n = 6, control + CQ n = 6, EGFRvIII n = 5, EGFRvIII + CQ n = 5).

To investigate the higher dependency of EGFRvIII-expressing cells on autophagy during metabolic stresses in vivo, control and EGFRvIII-expressing tumor xenografts were grown in nude mice (Fig. 3A) and treated with CQ (60 mg/kg daily). In line with previous results,^12^ EGFRvIII tumors displayed accelerated growth compared to control tumors (Fig. 3A). Interestingly, 7-d CQ treatment resulted in decreased growth (Fig. 3B; 20% increase in doubling time for control tumors and 30% increase for EGFRvIII expressing tumors, both p < 0.05). EGFRvIII expression was validated by immunohistochemistry (Fig. 3C).

CQ treatment changes the tumor microenvironment by decreasing the hypoxic fraction through reduced hypoxia tolerance^33^ and increased perfusion.^40^ Our in vitro data suggest that EGFRvIII-expressing cells were more dependent on autophagy for survival during hypoxia (Fig. 2). In line with the observed increased intrinsic tolerance of EGFRvIII-expressing cells to hypoxia (Fig. 2 and ref. 12), EGFRvIII-expressing tumors displayed a higher hypoxic fraction compared to control tumors as determined by pimonidazole immunohistochemistry (Fig. 3D). In both control tumors and EGFRvIII-expressing tumors CQ treatment resulted in a decrease in tumor hypoxia (Fig. 3D; p < 0.05 in control, p < 0.01 for EGFRvIII tumors), although this effect was most pronounced in EGFRvIII-expressing tumors.

As expected, cellular proliferation, as determined by BrdU incorporation, was higher in EGFRvIII-expressing tumors compared to control tumors (Fig. S2A), but was unaffected by CQ treatment, indicating that hypoxic cells contribute very little to proliferation in the tumor. In control tumors, CQ treatment increased vessel density (p < 0.05) and may have contributed to the reduction in tumor hypoxia (Fig. S2B). In contrast, EGFRvIII tumors showed a high vessel density when untreated, but there CQ treatment reduced, although to a limited extent (18%), the vessel density (p < 0.05). These results suggest that the effect of CQ on vessel function and abundance is phenotype dependent. No differences in perfused vessels in either of the tumors were observed (Fig. S2C). In contrast, the necrotic fraction increased significantly after CQ treatment in EGFRvIII-expressing tumors (2.3 fold, p = 0.01), but not in control tumors (Fig. 3E), suggesting that CQ treatment leads to cell death of EGFRvIII-expressing cells in vivo.

**Figure 4.**
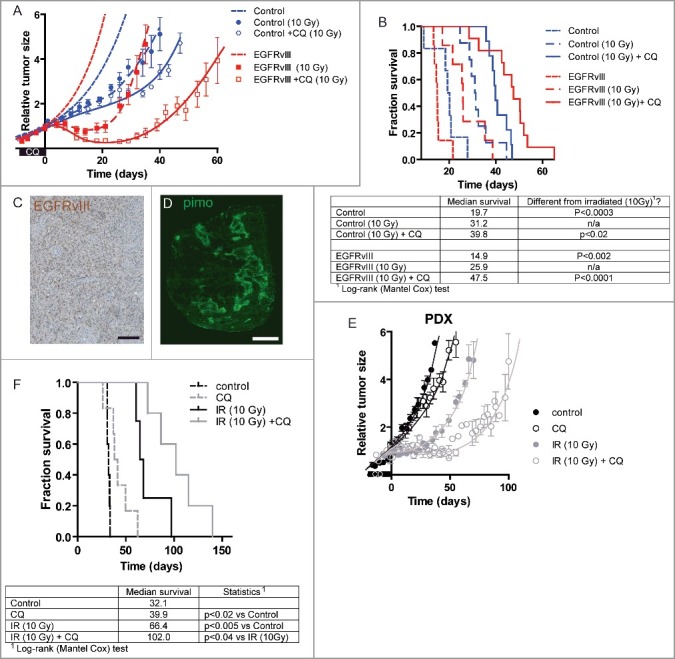
CQ treatment sensitizes EGFRvIII-expressing tumors to irradiation. (A) Growth curves of U373 xenografts: untreated (dashed lines; control [blue, n = 6], EGFRvIII[(red, n = 7]), treated with a single, tumor-specific dose of 10 Gy at t = 0 (interrupted line; control [blue, n = 8], EGFRvIII [red, n = 7]) or irradiated after 7 d CQ (60 mg/kg) pre-treatment from t = −7 to t = 0 and irradiated with a single, tumor-specific dose of 10 Gy at t = 0 (solid lines; control [blue, n = 9], EGFRvIII [red n = 11]). (B) Kaplan-Meier representations of the time to reach 4x the treated volume (from t = 0). Immunohistochemical staining for (C) EGFRvIII (scale bar: 50 μm) and (D) pimonidazole of PDX (scale bar: 2 mm), indicating EGFRvIII positivity and a substantial hypoxic fraction. (E) Growth curves of the PDX untreated (black closed circles, n = 5), treated with CQ (60 mg/kg) for 7 d from t = −7 to t = 0 (open circles, n = 5), treated with a single, tumor-specific dose, of 10 Gy at t = 0 (gray closed circles, n = 4) and treated with CQ (60 mg/kg) for 7 d from t = −7 to t = 0 followed by a single, tumor-specific dose, of 10 Gy at t = 0 (gray open circles, n = 5). (F) Kaplan-Meier representations of the time to reach 4x the treated volume (from t = 0).

Hypoxic cells contribute to radiotherapy resistance; we therefore determined whether the decrease in hypoxia after CQ administration could sensitize EGFRvIII-expressing tumors to irradiation. Control and EGFRvIII tumor bearing mice were treated with CQ for 7 d prior to irradiation and tumor regrowth was monitored. As expected, growth of control tumors was significantly delayed after a single, tumor specific, dose of 10 Gy and could be further delayed by CQ pre-treatment (Fig. 4A). EGFRvIII-expressing tumors respond faster to irradiation, often an indication of a different type of cell death^41^ but also regrow faster. Yet the effect of CQ pre-treatment on EGFRvIII-expressing tumors postponed regrowth even further (Fig. 4A). To illustrate the differences in radiation sensitivity of the individual tumors, the data are replotted in Fig. 4B as Kaplan–Meier plots. Tumor irradiation extended the median survival of mice with control tumors from 20 to 31 d, and CQ pre-treatment increased median survival further to 40 d. The effect of CQ addition in EGFRvIII-expressing tumors was much larger as irradiation extended survival from 15 to 25 d, but in combination with CQ pre-treatment this survival was extended to 48 d (Fig. 4B). This effect was even more pronounced after a larger irradiation dose (15 Gy), showing an additional increase from 61 to 108 d after CQ treatment prior to irradiation (Fig. S3).

Expression of EGFRvIII on GBM cells is rapidly lost when cultured in vitro, suggesting a survival and/or selection advantage for EGFRvIII-expressing cells in the tumor microenvironment. For this reason most studies on EGFRvIII are performed in established GBM cell lines in which EGFRvIII is ectopically expressed. These cells and xenografts might behave different than the original tumors as these cells are clearly not dependent on EGFRvIII signaling for survival or growth, and expression levels of EGFRvIII may be much higher than endogenous levels. We have therefore generated primary tumor xenografts directly derived from a cancer patient (patient derived xenograft; PDX), with an EGFRvIII-positive GBM. These primary tumor xenografts are thought to better resemble and reflect the original tumor and its response to therapy. After growth as a xenograft, EGFRvIII expression was retained over generations, as determined by EGFRvIII-specific immunohistochemistry (Fig. 4C) and a substantial hypoxic fraction was observed (Fig. 4D, mean ± Stdev 0.15 ± 0.04, n = 6). Similar to the ectopically EGFRvIII-expressing tumors, CQ administration reduced tumor growth (18 to 28 d doubling time p < 0.05) in endogenously expressing EGFRvIII-positive PDX (Fig. 4E), which resulted in an increase in median survival from 32 to 40 dayds (Fig. 4F). Tumor irradiation increased median survival to 66 d and could be increased by CQ administration to 102 d post treatment. Together, these data indicate that autophagy is a therapeutic vulnerability of EGFRvIII-expressing GBM that can be exploited by CQ administration.

### EGFRvIII-expressing cells are dependent on autophagy due to a high metabolic demand

**Figure 5.**
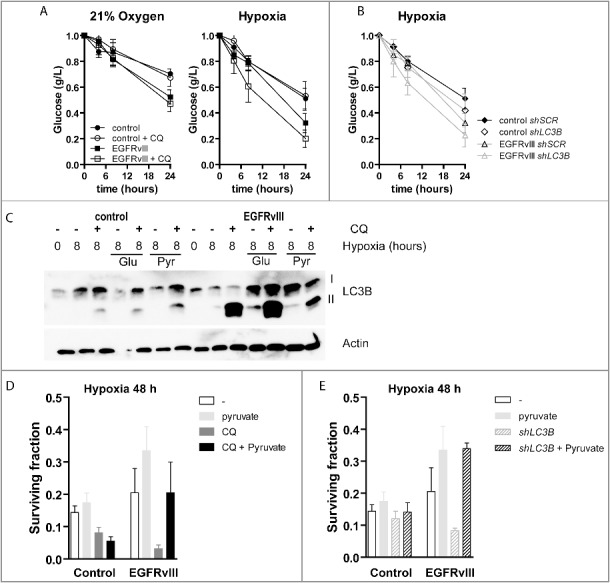
EGFRvIII-expressing cells require autophagy to meet high metabolic demand. (A) Glucose utilization by control and EGFRvIII-expressing U373 cells under ambient (left) or hypoxic (O_2_<0.02%) (right) conditions with or without addition of CQ (5 µg/ml). n = 3, mean ± SEM. (B) Glucose consumption after expression of a control shRNA or a shRNA targeting *LC3B*. n = 3, mean ± SEM (C) Autophagic flux determination in control and EGFRvIII-expressing cells after 8-h hypoxia exposure with additional glucose or methyl pyruvate supplementation (5 mM). (D) Clonogenic survival after 48 h hypoxia (O_2_<0.02%) exposure in the presence of CQ, methyl pyruvate or CQ and pyruvate. (O_2_<0.02%) n = 3, mean ± SEM. (E) Clonogenic survival after 48-h hypoxia (O_2_<0.02%) exposure in control and LC3B-depleted cells with pyruvate supplemented n = 3, mean ± SEM.

As EGFRvIII-expressing cells appear to be more dependent on autophagy for their survival during metabolic stress, we hypothesized that autophagy supports the high metabolic demand of EGFRvIII-expressing cells.^38^ We therefore assessed glucose abundance in the medium of control and EGFRvIII-expressing cells. Although glucose consumption was higher under normal culture conditions in EGFRvIII-expressing cells, the addition of CQ (5 µg/ml) did not change glucose consumption under ambient oxygen conditions (Fig. 5A, p < 0.05 at t = 24). As expected, glucose consumption increased during hypoxia (Fig. 5A). Interestingly, CQ addition did not change glucose consumption of control cells, but increased glucose consumption by EGFRvIII-expressing cells (Fig. 5A). Comparable to CQ, inhibition of autophagy through targeting *LC3B*, increased glucose consumption in EGFRvIII-expressing cells (Fig. 5B) during hypoxia, but also increased glucose consumption in control cells. This illustrates that besides autophagy, CQ and LC3B may also be involved or influence other processes.

The increased glucose metabolism after inhibition of autophagy during hypoxia in EGFRvIII cells, suggests that autophagy is used as an alternative mechanism required in support of the high metabolic demand and energy production. We therefore questioned whether the elevated autophagic flux in EGFRvIII cells during hypoxia is required to meet this demand. We therefore assessed autophagic flux after provision of additive external resources (glucose and pyruvate) for metabolic processing. In control cells, no changes in flux were observed after addition of glucose and/or pyruvate (Fig. 5C). In contrast, in EGFRvIII-expressing cells, addition of pyruvate, but not glucose, rescued the elevated autophagic flux. We hypothesized that failure to meet the high metabolic demand, for example through autophagy inhibition, results in cell death of EGFRvIII-expressing cells. To test this, we exposed control and EGFRvIII-expressing cells to hypoxia for 48 h in the presence or absence of CQ (Fig. 5D) or short hairpins targeting *LC3B* (Fig. 5E), supplemented the culture medium with additional pyruvate and assessed clonogenic survival. Interestingly, the addition of pyruvate rescued cell death in autophagy-deficient EGFRvIII-expressing cells (p < 0.01 in Fig. 5D and E), but not in control cells. This illustrates the therapeutic potential of targeting the high metabolic demand of EGFRvIII-expressing cells and tumors through inhibition of autophagy.

### Patients with EGFRvIII-positive GBM benefit from CQ treatment

**Figure 6.**
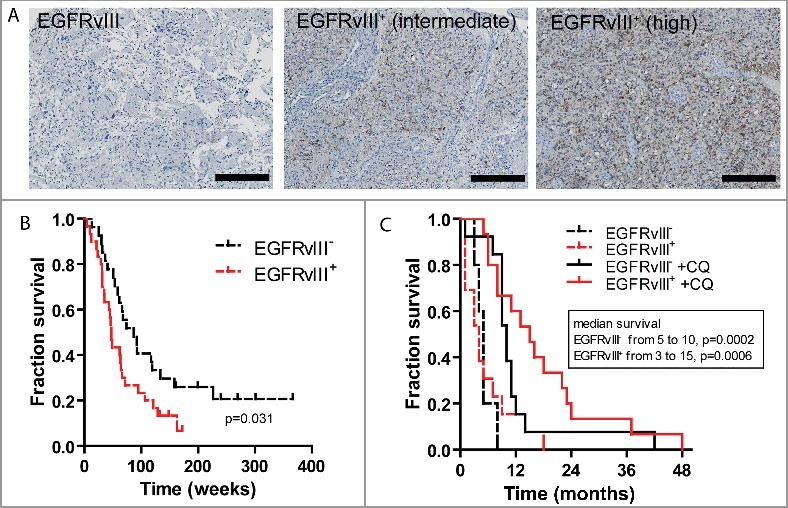
Patients with EGFRvIII-expressing GBM benefit most from concurrent CQ treatment. (A) Micrographs of immunohistochemically stained biopsy slides of a negative (left) and EGFRvIII-expressing tumors (middle and right). Scale bar: 100 μm. (B) Kaplan-Meier survival plots of 61 GBM patients after TMZ-radiotherapy treatment, separated based on EGFRvIII expression. (C) Survival plot of 43 GBM patients categorized for EGFRvIII status and CQ treatment. Patients with an EGFRvIII-positive tumor benefit substantially from CQ treatment.

Several reports have been published on the prognostic role of EGFRvIII in GBM patients. Although most reports indicate worse survival for EGFRvIII^+^ GBM patients, others have indicated a different outcome. This difference may depend on the method of EGFRvIII detection either through PCR or immunohistochemistry, or on the heterogeneity of the patient populations and therapeutic interventions included. We therefore selected 57 patients with a primary GBM, recently treated in our institution. In this cohort, 30 (53%) of the tumors were identified to express EGFRvIII through immunohistochemical analysis (Fig. 6A). All patients were treated with TMZ (75 mg per square meter of body-surface area per day) and radiotherapy (total dose 60 Gy, 2 fractions per day). In this very homogeneously treated patient cohort, immunohistochemical detection of EGFRvIII expression confirmed earlier reports^14^^,^^42^ which indicate significantly worse prognosis for EGFRvIII-expressing GBM patients (Fig. 6B, p < 0.05). Patient characteristics are listed in Table 1.

**Table 1.** Characteristics of GBM patients treated with TMZ and radiotherapy (60 Gy).

EGFRvIII status	EGFRvIII^−^	EGFRvIII^+^
	27 (47%)	30 (53%)
Male	15/27	19/30
Female	12/27	11/30
Karnofsky performance (pre-surgery)		
60 - 70	0 (0%)	2 (7%)
80 – 90	8 (30%)	19 (63%)
100	19 (70%)	9 (30%)
TMZ (75 mg/m^2^/d)	27/27 (100%)	30/30 (100%)
Radiotherapy (60 Gy)	27/27 (100%)	30/30 (100%)

To assess the clinical relevance of our findings in the treatment of EGFRvIII-expressing GBM, we retrospectively analyzed 43 GBM patients that were included in a randomized, placebo-controlled phase II trial.^43^ Of the analyzed patients, 28 patients received CQ in combination with carmustine and radiotherapy. EGFRvIII expression was assessed immunohistochemically and observed in 22 of 43 (51%) patients. Patient characteristics are listed in Table 2. Although the number of analyzed patients is low, these results are encouraging as CQ treatment improved median survival from 5 to 10 mo in patients with EGFRvIII-negative GBM (Fig. 6C, p = 0.0002). Strikingly, median survival of patients with EGFRvIII-positive GBM increased from 3 to 15 mo (p = 0.0006), confirming our pre-clinical findings and the therapeutic potential of CQ in the treatment of EGFRvIII-expressing tumors.

**Table 2.** Characteristics of GBM patients.

	no CQ treatment		CQ treatment	
EGFRvIII status	vIII^−^	vIII^+^	vIII^−^	vIII^+^
Male	5/8	3/7	10/13 (77%)	6/15 (40%)
Female	3/8	4/7	3/13 (23%)	9/15 (60%
Karnofsky performance (pre-surgery)				
< 70	0 (0%)	1 (14%)	0 (0%)	1 (7%)
70 – 80	7 (87%)	6 (86%)	7 (54%)	7 (47%)
90 −100	1 (13%)	0 (0%)	6 (46%)	7 (47%)
Karnofsky performance (post-surgery)				
< 70	1 (13%)	2 (29%)	0 (0%)	0 (0%)
70 – 80	1 (13%)	1 (14%)	1 (8%)	3 (20%)
90 −100	6 (74%)	4 (57%)	12 (92%)	12 (80%)
2nd surgery	4/8 (50%)	2/7 (29%)	2/13 (15%)	2/15 (13%)
Radiotherapy	3/8 (38%)	2/7 (29%)	8/13 (62%)	6/15 (40%)

## Discussion

Our data confirmed that the expression of EGFRvIII indicates a poor prognosis for GBM patients due to increased therapy resistance, increased proliferation and accelerated tumor growth. Furthermore, EGFRvIII-expressing cells are more resistant to metabolic stresses and result in an increased hypoxic fraction of the tumor. As tumor hypoxia is a major limiting factor in successful cancer treatment,^27^ targeting hypoxia in EGFRvIII-expressing tumors may therefore be essential in acquiring improved tumor control and ultimately tumor cure. Here we reveal that expression of EGFRvIII leads to increased autophagic flux during metabolic stresses such as hypoxia and starvation. In line with previous reports, EGFRvIII-expressing cells proliferate faster and display increased survival during these stresses.^10–13^ We show that these advantages can be abrogated through inhibition of autophagy either through genetic or pharmacological targeting. In vivo, EGFRvIII tumors display increased tumor growth and faster tumor regrowth after radiotherapy, effects that are likely to contribute to the poor outcome of EGFRvIII^+^ GBM patients. Importantly, CQ treatment inhibits the accelerated growth and regrowth of EGFRvIII tumors. As observed previously,^33^ CQ treatment results in reduced tumor hypoxia in tumor xenografts, yet we observed that the largest reduction occurs in EGFRvIII-expressing tumors. Strikingly, CQ treatment results in a drastic increase in necrosis of EGFRvIII tumors, but not in control tumors. In addition to lowering the intrinsic tolerance to hypoxia, CQ may induce vessel normalization and increase perfusion, resulting in decreased tumor hypoxia.^40^ In our experiments, vessel density increased in control tumors but not in EGFRvIII-expressing tumors (Fig. S2). Our data indicates that EGFRvIII expression changes the tumor cell dependence on autophagy to tolerate low oxygen levels. In the absence of autophagy, these cells will die leading to increased tumor necrosis. These results are in line with the enhanced cell death after autophagy inhibition in EGFRvIII-expressing cells, in vitro (Fig. 2C and D).

During autophagy, recycled products are transported across the autolysosomal membrane into the cytoplasm. These metabolites can be reused in protein and ATP production.^34^ In our studies we observed that EGFRvIII^+^ tumors cells displayed increased activation and dependence on autophagy during hypoxia, effects that can be rescued by adding pyruvate to the culture medium. As EGFRvIII^+^ cells consume more glucose (Fig. 5), autophagy may be essential in supporting metabolic processes in EGFRvIII^+^ cells. EGFRvIII may induce the increased glucose uptake and consumption through enhanced RRAS signaling. Similar to our observations, oncogenic RRAS promotes glucose uptake and utilization in glycolysis (reviewed in ref. 44). Interestingly, the effect of autophagy inhibition in EGFRvIII^+^ cells on survival and glucose consumption during hypoxia could not be rescued by glucose addition, but was rescued after pyruvate supplementation. This suggests that a) the glycolytic pathway is used to its fullest extent, and b) the high metabolic activity is not used to support ATP production, as pyruvate cannot further be degraded in the citric acid cycle due to the absence of oxygen. In addition to being converted to ATP, pyruvate is able to prevent toxic effects of oxygen radicals and peroxides and indirectly prevents the depletion of glutathione during hypoxia,^45^ and may therefore decrease the toxic effects after reoxygenation.

From a clinical perspective, the most important finding of this research is the relatively large effect of CQ on tumors that express EGFRvIII compared to the non-EGFRvIII-expressing tumors. In cell line-derived xenografts, we observed transition of the hypoxic into necrotic areas and a high degree of sensitization to radiotherapy. These results were further confirmed in patient-derived xenografts. Furthermore, retrospective analysis of EGFRvIII expression in GBM patients treated with carmustine, radiotherapy and surgery revealed a larger improvement in survival for patients with EGFRvIII-positive GBM after concurrent CQ treatment compared to patients with EGFRvIII-negative tumors. Although these initial clinical results are promising, larger cohorts of patients are required to fully understand the therapeutic potential of CQ administration in the treatment of GBM. Recently a phase I/II trial in newly diagnosed GBM has been reported where standard of care (temozolomide and radiotherapy) was combined with concurrent hydroxychloroquine (a derivative of CQ).^46^ Due to unfavorable toxicity profiles and dose-limiting toxicity before consistent inhibition of autophagy was observed, no changes, compared to historical data (EORTC trial), in overall survival were observed. Although hydroxychloroquine and chloroquine belong to the same class of 4-aminoquinolines, their effective doses and toxicity profiles differ. Clinical trials to evaluate toxicity and effectiveness (randomized phase II trial) of CQ in combination with temozolimide and radiotherapy are ongoing in our institute (clinicaltrials.gov identifier NCT02378532 and NCT02432417). Based on the findings described in this manuscript, in the phase II trial patients will be stratified based on expression of EGFRvIII.

Interestingly, other mutation types in the RAS-RAF-MAPK/ERK pathway have also been associated with increased dependence on autophagy for survival. For example, HRAS^G12V^ or KRAS^G12V^ transformed cells upregulate their basal autophagy and depend on elevated autophagy for survival during starvation.^47^ Also the BRAF^V600E^ mutation, which occurs in over 50% of childhood central nervous system tumors, results in elevated autophagy and increased sensitivity to pharmacological or genetic autophagy inhibition in terms of cell viability.^48^ Remarkable clinical responses have been observed.

These tumors are often also effectively treated using kinase inhibitors, such as vemurafenib. Unfortunately, development of resistance is often observed. Levy et al. reported that autophagy contributes to vemurafenib resistance. Hence, targeting autophagy can overcome this resistance, and patients with resistant tumors display favorable clinical responses when chloroquine is combined with vemurafenib.^49^ Combined with our data these results suggest that constitutive signaling through the RAS-RAF-MAPK/ERK pathway creates autophagy dependence in tumor cells. This important information could be used for individualized treatment selection for patients with brain tumors.

In conclusion, our results demonstrate that elevated autophagy activity contributes to the enhanced tolerance to metabolic stresses of EGFRvIII-expressing cells. Targeting this survival mechanism abrogates this advantage and results in enhanced tumor cell killing. Ultimately this downregulation of autophagy activity leads to reduced tumor growth, reduced tumor hypoxia, increased tumor necrosis and sensitization of tumors to radiotherapy. In line with these pre-clinical findings, we observed that patients with EGFRvIII-expressing GBM benefit most from concurrent CQ treatment. Concurrent CQ treatment in combination with conventional therapy seems therefore a promising strategy to improve the outcome for EGFRvIII^+^ GBM patients.

## Materials and methods

### In vivo experiments

Animal facilities and experiments were in accordance with local institutional guidelines and approved by the local animal welfare committee. Experiments were performed as described previously.^33^ Briefly, tumors were grown in NMRI-nu (nu/nu) female mice (Charles River). Cells in matrigel (BD Biosciences, 354234) were injected subcutaneously (1.5 × 10^6^ cells). For the generation of primary human xenografts, a small piece of biopsy (approx. 1 mm^3^) was implanted for the generation of the first generation tumor (growth until 1 cm^3^). This tumor was cut into multiple pieces, implanted in recipient mice and experiments were subsequently performed on these 2^nd^ generation tumors. Tumor size was assessed by caliper measurement in 3 orthogonal diameters. Mice treated with CQ (Sigma Aldrich, C6628) received 60 mg/kg CQ for 7 consecutive d. For radiation treatment, tumors were positioned in the irradiation field using a custom-built jig and irradiated with a single dose of 10 Gy (15 MeV e-) using a linear accelerator (Varian, Palo Alto, CA, USA)^50^ Number of animals used are listed in the respective figure legend.

### Patient studies

The retrospective analyses and the establishment of patient derived xenografts were approved by the respective local medical ethics committees. All patients with a primary GBM, treated in Maastricht with radiotherapy between 2007 and 2012 with sufficient histological tissue available and who had completed the planned treatment with temozolomide (75 mg/m^2^/day) and radiotherapy (60 Gy), were included in the analysis (Fig. 6B and Table 1). The effect of CQ on prognosis in EGFRvIII-expressing GBM was evaluated on 43 GBM patients (of which sufficient tumor tissue was still available) that were included in a randomized, placebo controlled phase II trial (Fig. 6C and Table 2).^43^

### Reagents and cell lines

Unless specified otherwise, all reagents were obtained from Sigma Chemical Co. (the Netherlands) and all electrophoresis reagents were from Bio-Rad (the Netherlands). MEM-α medium was obtained from Invitrogen (10370070). Glutamax-I (35050038) and pyruvate (11360039) were obtained from Life Technologies, DMEM (BE12-604F) and fetal bovine serum (FBS) were from GE-Healthcare. Glucose determination kits were purchased from Biosentec (010). Cell lines were obtained from and maintained as described by ATCC (HTB-14 (U87) and HTB-17 (U373)), negative for mycoplasma contamination (Lonza Mycoalerts, LT07-318). The cells’ origin was authenticated by STR analysis (Identicell). Cell lines that endogenously express EGFRvIII (DKMG and BS153) were kindly provided by Malte Kriegs and Nina Struve (Hamburg, Germany) and maintained as described previously.^39^ For doxycycline (Sigma, D9891) inducible knockdown of *LC3B*, shRNA [TTTCTCACTCTCATACACCTCT] (Sigma Aldrich, TRCN0000152696) was expressed from Tet-pLKO-puro (addgene, 21915; depositor Dmitri Wiederschain^51^) as described previously.^52^ *ATG7* knockdown was induced using pTRIPZ lentiviral delivery of sh*ATG7* [ATACAGTGTTCCAATAGCTGGG] (Dharmacon). Dominant negative ULK1^K46I^ was used as described previously.^53^ Differences in autophagic flux were confirmed using mCherry-EGFP-LC3 expression from a pBabe-puro vector (addgene, 22418; depositor J. Debnath).^54^

### Western blotting

Cells were lysed and processed as described previously^7^ and proteins were separated through SDS-PAGE. After transfer, proteins were probed with antibodies against EGFR, EGFRvIII runs at lower height (Santa Cruz Biotechnology, sc-03), MAP1LC3B (Cell Signaling Technology, 2775S), ACTB/actin (MP Biomedicals, 08961001), p-EGFRvIII (Cell Signaling Technology, 3777), p-MAPK/ERK (Cell Signaling, 4370) or tubulin (Cell Signaling Technology, 2146). Bound antibodies were visualized using HRP-linked anti-rabbit (Cell Signaling Technology, 7074S) or anti-mouse (Cell Signaling Technology, 7076S) antibodies.

### Immunofluorescence and immunohistochemistry

For immunofluorescence, cells were grown on glass coverslips (VWR, 470019-016). After exposure, cells were washed and fixed in 4% paraformaldehyde (Sigma Aldrich, p6148). After permeabilization, cells were probed with anti-MAP1LC3B (Cell Signaling Technology, 2775S) followed by anti-rabbit Alexa Fluor 488 (Invitrogen, A11006). For EGFRvIII expression determination in tumors, formalin-fixed paraffin-embedded tissue slides were probed with antibodies directed against EGFRvIII (clone L8A4, kindly provided by Dr. Bigner [Duke University Medical Center, Durham, NC]).

For assessment of microenvironmental changes in the xenograft studies, frozen, acetone-fixed sections were stained by using anti-pimonidazole (Hypoxyprobe Inc, HP4-100kit), 9F1 (rat monoclonal antibody to mouse endothelium, Department of Pathology, Radboud University Medical Center) or anti-BrdU (Sigma-Aldrich, B8434; as described previously^33^). For quantitative analysis, the slides were scanned with a computerized digital image processing system by using a high-resolution intensified solid-state camera on a fluorescence microscope (Axioskop; Zeiss, the Netherlands) with a computer-controlled motorized stepping stage.^33^

### Proliferation assessment

Cells were seeded in a 24-well culture plate (Greiner Bio One, 662160) and incubated under normal, serum starved or hypoxic conditions (0.2% O_2_). CQ was used at a concentration of 5 µg/ml (Sigma Aldrich, C6628). Cellular density was determined using Cristal violet staining.

### Clonogenic survival assay

Eight h after counting and seeding, cells were exposed to serum-free medium or hypoxia (O_2_ <  0.02%, Don Whitley, VA500) as described previously.^55^ After exposure the medium was replaced with normal medium and cells were incubated under standard culture conditions until colonies formed (10 d). Colonies were visualized using 0.4% methylene blue in 70% ethanol staining. Only colonies consisting of ≥50 cells were counted.

### Statistics

Statistical analyses were performed using graphpad software. When comparing 2 conditions unpaired *t* tests were used. For multiple comparisons, one way-ANOVA with Bonferroni corrections were used. p-values <  0.05 were considered statistically significant.

## Supplementary Material

supp-data_1409926.zip
