# Supplementary material for: EGFRvIII expression triggers a metabolic dependency and therapeutic vulnerability sensitive to autophagy inhibition
Source: Autophagy. 2018 Jan 29;14(2):283–95. doi: 10.1080/15548627.2017.1409926 (PMC5902239; doi:10.1080/15548627.2017.1409926)
Supplement: supp-data_1409926.zip [file kaup-14-02-1409926-s001.zip › supp-data_1409926/2017AUTO0037R2-s02_1409926.docx]

**Figure S1.** Increased autophagic flux is EGFR activity dependent. (**A**) Autophagic flux determination in the presence of afatinib in increasing concentrations (0, 10, 25, 50 and 100 nM). p-EGFRvIII, and p-MAPK/ERK are included to confirm afatinib effectivity. (**B**) Growth of BS153 and (**C**) DKMG cells in the presence of 5 µg/ml CQ.

**Figure S2.** Tumor microenvironmental factors after CQ treatment. BrdU labeling index (**A**), vessel density (**B**) and perfused vessel fraction (**C**) in control and EGFRvIII-expressing tumors, when indicated, treated with CQ for 7 days. ev, empty vector.

**Figure S3.** CQ treatment increases growth delay after irradiation. Time for EGFRvIII-expressing U373 xenografts to regrow to 4 times the irradiated volume after a single dose irradiation with 15 Gy. When indicated, animals were treated for 7 consecutive days with 60 mg/kg CQ prior to irradiation (n=7 per group).
