# Supplementary figures and images for: EGFRvIII expression triggers a metabolic dependency and therapeutic vulnerability sensitive to autophagy inhibition
Source: Autophagy. 2018 Jan 29;14(2):283–95. doi: 10.1080/15548627.2017.1409926 (PMC5902239; doi:10.1080/15548627.2017.1409926)

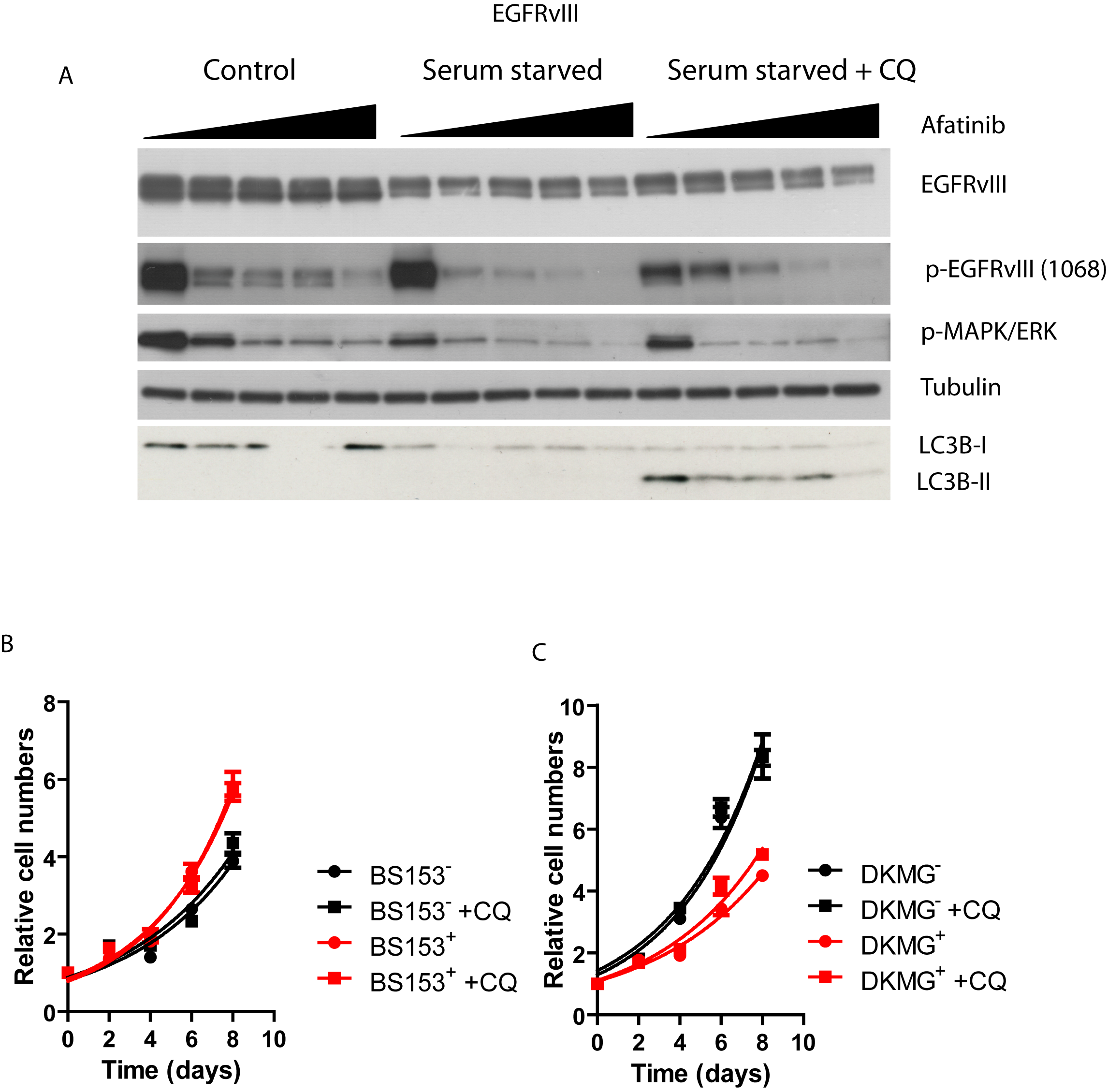

Supplement: supp-data_1409926.zip [file kaup-14-02-1409926-s001.zip › supp-data_1409926/2017AUTO0037R2-s03_1409926.tif]

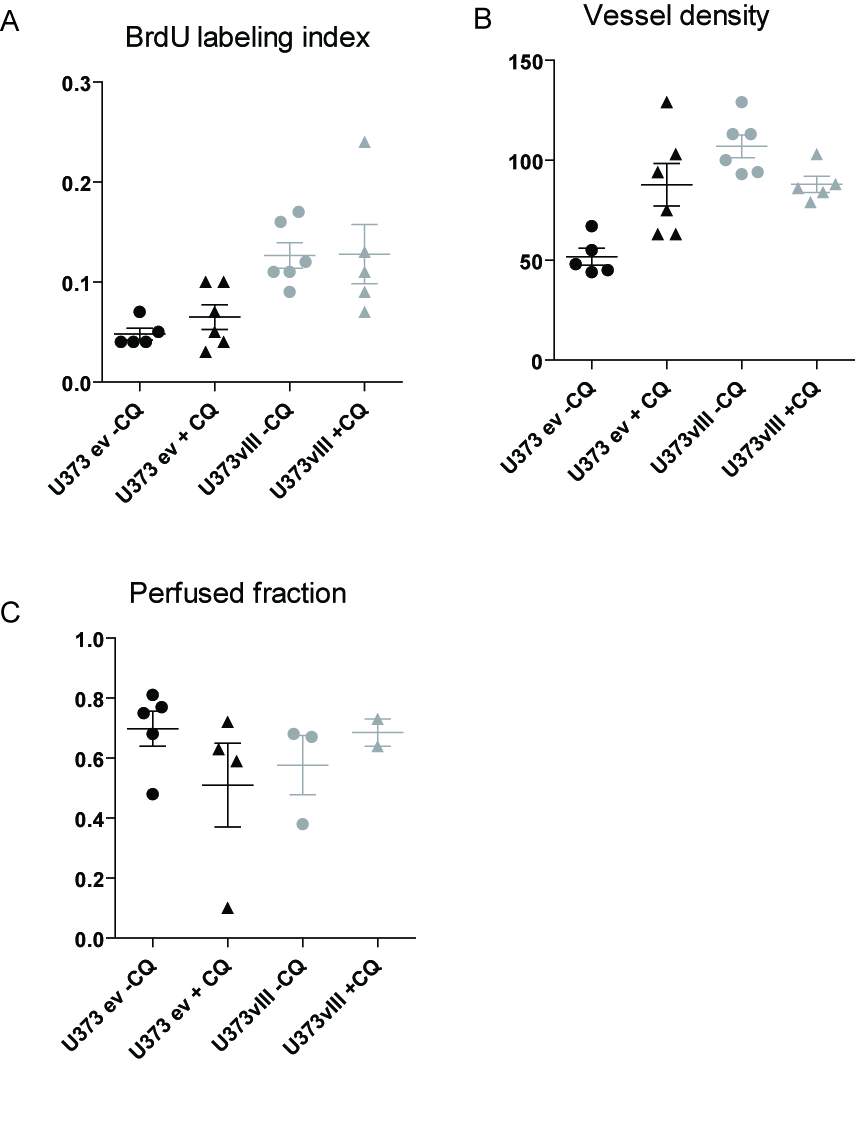

Supplement: supp-data_1409926.zip [file kaup-14-02-1409926-s001.zip › supp-data_1409926/2017AUTO0037R2-s04_1409926.tif]

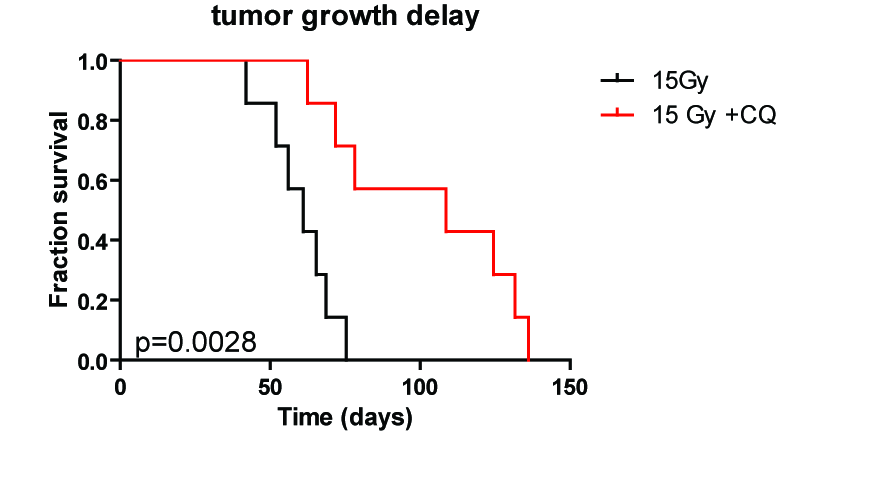

Supplement: supp-data_1409926.zip [file kaup-14-02-1409926-s001.zip › supp-data_1409926/2017AUTO0037R2-s05_1409926.tif]
